# Superior mesenteric artery syndrome following initiation of cisplatin-containing chemotherapy: a case report

**DOI:** 10.1186/1752-1947-6-14

**Published:** 2012-01-16

**Authors:** Atsuhito Ushiki, Tomonobu Koizumi, Hiroshi Yamamoto, Masayuki Hanaoka, Keishi Kubo, Mina Matsushita

**Affiliations:** 1 First Department of Internal Medicine, Shinshu University School of Medicine, 3-1-1, Asahi, Matsumoto, 390-8621, Japan; 2 Department of Radiology, Shinshu University School of Medicine, Matsumoto, 390-8621, Japan

**Keywords:** superior mesenteric artery syndrome, body weight loss, emesis, non-small cell lung cancer

## Abstract

**Introduction:**

Superior mesenteric artery syndrome is a rare cause of upper intestinal obstruction resulting from compression of the duodenum by the superior mesenteric artery and abdominal aorta.

**Case presentation:**

We describe a case of superior mesenteric artery syndrome in a 61-year-old Japanese man with non-small cell lung cancer who had been treated with cisplatin-containing chemotherapy and had lost 7 kg in weight. The diagnosis was confirmed by the typical findings of abdominal computed tomography showing distended stomach resulting from compression of the third portion of the duodenum and reduction of an aortomesenteric distance and aortomesenteric angle.

**Conclusions:**

This case highlights the importance of considering the possibility of superior mesenteric artery syndrome in patients treated with chemotherapy, especially those presenting with a low body mass index and showing weight loss during chemotherapy.

## Introduction

Superior mesenteric artery (SMA) syndrome, also known as Wilkie's syndrome, is a rare cause of upper gastrointestinal obstruction. In the SMA syndrome, the third portion of the duodenum is trapped between the abdominal aorta and the SMA. The clinical symptoms include postprandial epigastric pain, nausea, vomiting, anorexia, and weight loss due to duodenal obstruction [1]. SMA syndrome is directly related to anatomical and mechanical factors. In contrast to the position of four-legged mammals, the aortomesenteric angle in humans changes from 90° to an accentuated acute angle, leading to vascular constriction at the location where the duodenum usually crosses thereby triggering the syndrome. In addition, the development of SMA syndrome is generally associated with acute or chronic reduction of retroperitoneal fat [2]. Numerous predisposing conditions for SMA syndrome, such as cancer, trauma, anorexia nervosa, and postoperative states, have been identified with potential impacts on the aortomesenteric angle. However, SMA syndrome has rarely been reported in patients treated with systemic chemotherapy. We report the case of a patient with non-small cell lung cancer (NSCLC) treated with cisplatin-containing chemotherapy who developed typical clinical and radiological findings of duodenal obstruction due to SMA syndrome. The patient experienced weight loss because of severe emesis after the initiation of chemotherapy. Our experience suggests that SMA syndrome could be present in patients receiving systemic chemotherapy.

### Case Presentation

**Figure 1:**
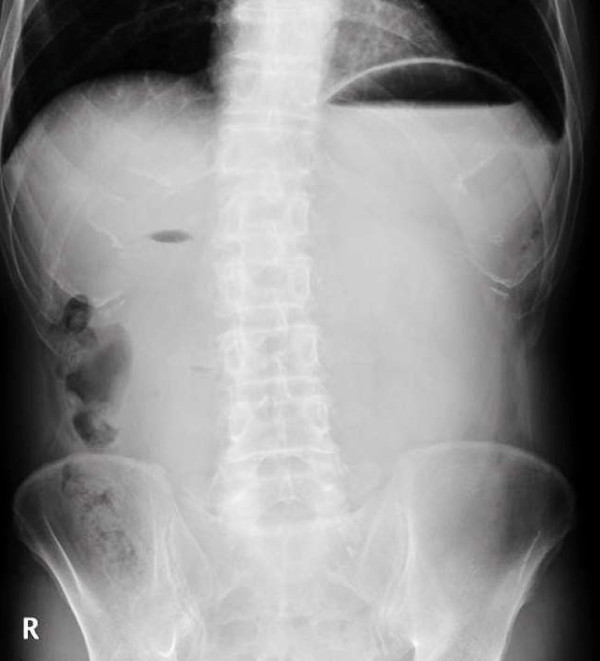
**Abdominal X-ray showed distension sign of the stomach with air fluid level and duodenal gas**.

**Figure 2:**
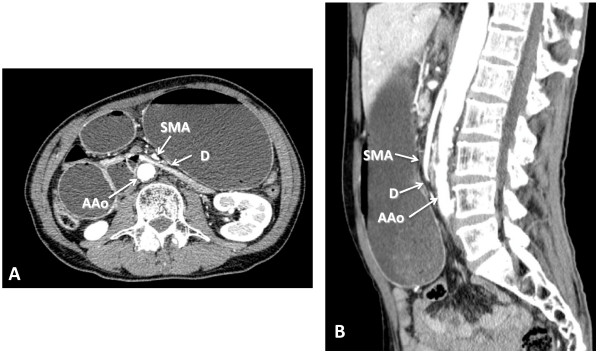
**Abdominal computed tomography**. Abdominal computed tomography showed distension of the stomach and the duodenal bulb due to compression of the third portion of duodenum. The distance between the abdominal aorta and superior mesenteric artery was 6.1 mm **(A)**. The angle between the abdominal aorta and superior mesenteric artery was 15.8° **(B)**. AAo, abdominal aorta; D, duodenum; SMA, superior mesenteric artery.

A 61-year-old Japanese man with weight 49.6 kg and height 1.68 m (body mass index (BMI) = 17.6 kg/m^2^) was diagnosed with stage IIIB squamous cell carcinoma of the lung in March 2009. He lost 3 kg in body weight over a period of six months. He underwent one course of systemic chemotherapy (cisplatin at 80 mg/m^2 ^and docetaxel at 60 mg/m^2 ^on day one). He was treated prophylactically with dexamethasone and granisetron for antiemesis just before the chemotherapy. From day three to day 10 after the initiation of chemotherapy, he had anorexia, nausea and vomiting, estimated as grade three according to the common terminology criteria for adverse event, Version 4, and lost 4 kg in weight. On day 17, he presented with epigastric pain and vomiting. Physical examination showed a distended abdomen and decreased bowel sound. Plain abdominal X-ray showed a distended stomach and duodenal gas (Figure 1). Contrast-enhanced abdominal computed tomography (CT) showed a distended stomach and duodenal bulb due to compression of the third portion of the duodenum, an aortomesenteric distance of 6.1 mm (normal: 10 to 28 mm) and a reduction of the aortomesenteric angle to 15.8° (normal: 25° to 60°) (Figure 2) [3]. These findings were highly suggestive of SMA syndrome.

**Figure 3:**
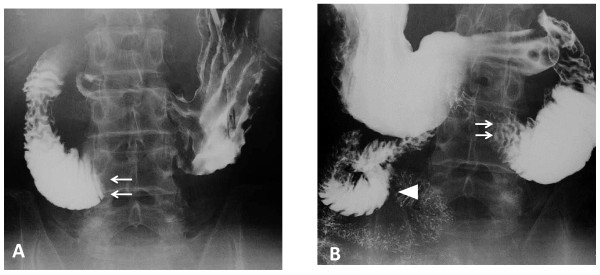
**Upper gastrointestinal series**. Upper gastrointestinal series in the supine position showed an abrupt cut-off (arrows) at the third portion of the duodenum **(A)**. In the prone position, the contrast medium passed through the obstructed part (arrows) of the distal side (arrowheads) **(B)**.

An upper gastrointestinal series in the supine position showed a sharp cut-off at the third portion of the duodenum (Figure 3A). In the prone position, the contrast medium passed through the obstructed part of the distal side of the third portion of duodenum (Figure 3B). After inserting a nasogastric tube, more than 2700 mL turbid, green fluid was drained. His symptoms improved after nasogastric tube drainage and he remained in the prone position. The patient was given total parenteral nutrition for 10 days and tubal feeding for the following 10 days. After 20 days, his body weight increased from 45.6 to 49.0 kg and he returned to oral intake without subsequent symptoms. Subsequent chemotherapy using the same regimen was started after his body weight reached more than 50 kg. He presented with grade 2 emesis during chemotherapy but developed neither SMA syndrome nor weight loss. A total of four cycles of chemotherapy were performed, and the patient achieved a partial response.

## Discussion

The clinical manifestation of a compression of the third portion of the duodenum by SMA was initially described by Rokitansky *et al. *in 1861 [4]. Subsequently, Wilkie reported seven instances of SMA syndrome in 1927 [5] and his name has become a common eponym for the SMA syndrome.

A number of case reports and several reviews have been reported [1,2,6-11]. In general, most cases of SMA syndrome in patients with neoplasms were due to mechanical compression by intraabdominal tumor. However, severe weight loss results in reduction of the mesenteric fat pad, which can lead to compression of the duodenum by the SMA. Cases of SMA syndrome due to severe weight loss, such as trauma, eating disorders, rheumatoid disease, cardiac cachexia, human immunodeficiency virus infection, and diabetes mellitus, have been reported [6-11]. Neoplasms and systemic chemotherapy can induce anorexia and body weight loss. Our case had a low BMI before chemotherapy and showed a further reduction in body weight after the initiation of chemotherapy. Our search of the English medical literature revealed no reports of SMA syndrome in patients receiving chemotherapy. To the best of our knowledge, this is the first proven case of SMA syndrome precipitated by weight loss due to NSCLC and chemotherapy. It is well known that chemotherapy using cisplatin stimulates gastroduodenal vagal afferent nerves and is frequently associated with emesis. We would like to emphasize that SMA syndrome is a rare but noteworthy complication in cancer patients, especially those presenting with severe emesis and weight loss during chemotherapy. On the other hand, there are several reports documenting mesenteric vascular complications during systemic chemotherapy using cisplatin [12,13]. Allerton described a case of acute mesenteric ischemia after chemotherapy using cisplatin [14]. Therefore, we have to distinguish SMA syndrome from acute mesenteric ischemia if a patient complains of abdominal pain.

In patients with symptoms suggesting SMA syndrome, further radiographic studies should be performed to establish the diagnosis. The following strict radiographic criteria have been established for diagnosis of SMA syndrome: (i) dilatation of the first and second portions of the duodenum with or without gastric dilatation, (ii) abrupt vertical and oblique compression of the mucosal folds, (iii) antiperistaltic flow of contrast medium proximal to the obstruction, (iv) delay in transit of four to six hours through the gastroduodenal region, and (v) relief of obstruction in the prone, knee-chest, or left lateral decubitus position [1]. The classic diagnostic procedure is an upper gastrointestinal series. In patients with SMA syndrome, upper gastrointestinal series shows duodenal dilatation, retention of barium within the duodenum, and characteristic vertical linear extrinsic pressure on the third portion of the duodenum, but these findings are nonspecific for the SMA syndrome [3].

Contrast-enhanced computed tomography (CT) scan shows the advantage of providing an overall assessment of the abdominal cavity. This is the best way to delineate anatomical relationships and has been shown to be an effective and noninvasive method for evaluating the aortomesenteric angle, distance, fatty tissue, obstruction of the duodenum, and potential culprits for compression [15]. In normal individuals, the aortomesenteric angle was reported to be 25° to 60° and the aortomesenteric distance was reported to be 10 to 28 mm [3]. On the other hand, subjects presenting with an aortomesenteric angle of < 22° to 25° and distance of < 8 mm were affected by SMA syndrome [16]. Cut-off values of SMA syndrome were reported to be aortomesenteric angle of 22° (42.8% sensitivity and 100% specificity) and aortomesenteric distance of 8 mm (100% sensitivity and specificity) [17]. The measurement of the aortomesenteric angle (15.8°) and aortomesenteric distance (6.1 mm) in our case were consistent with the criteria for SMA syndrome.

Initial treatment of SMA syndrome usually involves a conservative approach. Nasogastric drainage for gastric decompression and mobilization into the prone or left lateral decubitus position are effective in the acute setting [1]. Both enteral jejunal feeding and total parenteral nutrition have been useful for increases in body weight promoting restoration of the retroperitoneal fat tissue with consecutive increases in the aortomesenteric angle and distance [1,18]. Surgical treatments, including open or laparoscopic duodenojejunostomy or duodenal mobilization and division of the ligament of Treitz, are normally indicated in symptomatic patients when conservative treatment fails [1].

## Conclusions

To the best of our knowledge, this is the first report of SMA syndrome during cisplatin-containing chemotherapy. Our case highlights the importance of considering the possibility of SMA syndrome in patients treated by chemotherapy, especially those presenting with low BMI and showing weight loss during chemotherapy. Contrast-enhanced computed tomography (CT) is a reliable and noninvasive tool for diagnosis of SMA syndrome. Total parenteral nutrition and tubal feeding with the aim of increasing body weight are useful forms of treatment in such cases.

## Consent

Written informed consent was obtained from the patient for publication of this case report and any accompanying images. A copy of the written consent is available for review by the Editor-in-Chief of this journal.

## Competing interests

The authors declare that they have no competing interests.

## Authors' contributions

AU and TK analyzed and interpreted the patient data and wrote the manuscript. HY, MH and KK analyzed and interpreted the patient data. MM interpreted the radiological radiograms.

All authors have read and approved the final manuscript.
